# Finite-Time Thermodynamics in Economics

**DOI:** 10.3390/e22080891

**Published:** 2020-08-13

**Authors:** Anatoly Tsirlin, Larisa Gagarina

**Affiliations:** 1 Ailamazyan Program Systems Institute of Russian Academy of Sciences, 152120 Rostov, Russia; 2 Institute of Systems and Program Engineering and Information Technologies, National Research University of Electronic Technology, 124482 Zelenograd, Russia; gagar@bk.ru

**Keywords:** thermodynamics, economics, optimal processes, irreversibility

## Abstract

In this paper, we consider optimal trading processes in economic systems. The analysis is based on accounting for irreversibility factors using the wealth function concept. The existence of the welfare function is proved, the concept of capital dissipation is introduced as a measure of the irreversibility of processes in the microeconomic system, and the economic balances are recorded, including capital dissipation. Problems in the form of kinetic equations leading to given conditions of minimal dissipation are considered.

## 1. Introduction

Systems that include a large number individually unobservable and uncontrollable elements which interact with each other are called *macrosystems*. The behavior of a component in such a system can be stochastic and yet the behavior of the system on a macro level, when averaged processes are observed, is deterministic. Thermodynamic systems of various nature with a large number of molecules interacting with each other on a micro level are a classical example of a macrosystem.

When contact is established between inhomogeneous macrosystems the processes of stochastic interaction occur. In thermodynamics these are, e.g., heat exchange, diffusion, and chemical transformation processes. It is not possible to return the system, when stochastic interaction process occurred, into its initial state without changing the system’s environment. This *irreversibility* of spontaneous processes of stochastic interactions is the key feature of macrosystems.

Microeconomics studies interaction of economic agents (EAs). An EA is a group of individual agents whose averaged characteristics determine the EAs’ characteristics. Sometimes we will use an analogy between microeconomics and thermodynamics and refer to the economic system (ES), where all economic agents are subsystems. The interaction between EAs leads to exchange of resources between them and consumption and/or production of these resources by them. In the course of these interactions each agent strives to increase its utility by choosing which kind of resource to exchange with which other kind of resource and in which quantity. Economic systems can be *isolated* from the environment. In this case all the exchange takes place inside the system. Economic systems can be *open*. Then, exchange of all or some of the resources can also occur between the system and its environment.

The processes of stochastic interactions in economics are irreversible as they are in any macrosystem. However, they are quite different from irreversible processes in thermodynamics chiefly because each subsystem chooses to participate in an exchange if that does not lead to a “loss”. Nevertheless, it is also possible to define an economic measure of irreversibility that attains maximum for an isolated system in equilibrium (like entropy in thermodynamics). It is also possible to define a non-negative function in economics similar to entropy production in thermodynamics and to formulate economic balances that include this function.

In the case when the duration of the processes is limited or the average intensity of the flows is fixed in the economy, the situation is very similar to finite-time thermodynamics (FTT).

In this paper a macrosystem approach to economic systems modeling is described. A number of economic problems that are similar to classical thermodynamic problems are solved.

## 2. Major Types of Economic Agents and Their Characteristics

The state of an economic agent is described by the vector of its stocks (amount of holdings) of resources $N=N_{1},N_{2},\ldots ,N_{k}$ and capital (cash) M. We assume that capital is measured by all economic agents using a single common unit (e.g., gold or an international currency). N and M are extensive variables, that is, when homogeneous economic systems merge/split, the values of these variables change in the same proportion. An economic system is also described by a vector of intensive variables—the estimates of how valuable these resources are for it given by the prices $p=(p_{1},\ldots p_{k})$ and the estimate of how valuable capital is for this system $p_{0}$. When economic systems merge, these variables equalize. *The new estimate $p_{i}$ for the resource i (its internal equilibrium price) is equal to the minimal price, in units of capital, for which the economic agent is prepared to sell resource i and the maximal price at which it is prepared to buy it*.

When an economic agent is offering to buy and sell resources, it is described by its supply and demand functions. The demand function shows the quantity of the i-th resource it is prepared to purchase for the price $c_{i}$. The higher this price is, the lower, as a rule, is this demand. Finally, at some price $c_{i}=p_{i}$ the economic agent stops buying. This is similar to the dependence of the heat flux on the temperature of the source and the working fluid in a heat engine. If $c_{i}>p_{i}$, then it is prepared to sell the i-th resource, and the higher $c_{i}$ is the larger quantity it is prepared to sell.

The unit of $p_{i}$ is the unit of M divided by unit of $N_{i}$. These estimates are related to amounts of resources and capital of an economic agent in the same way intensive variable in thermodynamics are related to extensive ones. The units of $c_{i}$ and $p_{i}$ are the same, but $c_{i}$ could be set manually by some intermediary agent. The estimate $p_{0}$ is the value of capital in units of some basic currency, for example, gold. More detailed discussion of the relationship between estimate of some resource and capital is given below.

In many cases demand and supply functions relate the price not to the quantity but to the flow of resource, $n_{i}\left(p_{i},c_{i}\right)$. The function $n_{i}\left(p_{i},c_{i}\right)$ determines the kinetics of resource exchange. If we define positive flow directed toward the economic agent then

(1) $$\begin{array}{c} \mathrm{sign}\;n_{i}\left(c_{i},p_{i}\right)=\mathrm{sign}\;\left(p_{i}-c_{i}\right)\\ \;n_{i}\left(c_{i},p_{i}\right)=0\;\mathrm{when}\;c_{i}=p_{i}\;\mathrm{and}\;\frac{\partial n_{i}}{\partial c_{i}}<0. \end{array}$$

The dimension of the vector c is the unit of capital divided by the unit of resource.

We define three types of economic agents.

1. *Economic agents whose resource estimates $p_{i}$ depend on the agent’s state* (on its stocks of resources and capital). Usually, but not always, when the stock of a resource is decreasing its estimate is decreasing too, and when capital is increasing then the estimate is increasing. The economic agent can also exchange capital M with the environment. Here, the minimal price of selling (maximal price of buying) is the economic agent’s estimate of capital. We denote it as $p_{0}\left(N,M\right)$. We shall call such systems *economic systems with finite capacity*.
2. *Economic agents with estimates $p_{i}$ independent of stocks of the resource* are similar to thermodynamic systems with infinite capacity (reservoirs). We shall call them *economic reservoirs*. Economic markets where prices do not depend on the rate of trading are examples of economic reservoirs. The amount of resource that is sold/purchased here is so small in comparison with its stock that in practice it does not effect its estimate.
  In the general case, a market’s demand/supply function n(c,p) depends on the prices of selling (buying) and on the estimates, and it obeys the conditions (1) for resource exchange kinetics. Such a market is called *monopolistic*. In the limit when for each flow n the difference between the price and estimate is infinitesimal (prices for any rate of flow n are equal to the market estimates), then the market is called a *market with perfect competition*. If this market is a reservoir then its prices do not depend on the demand but change over time under the influence of the external system factors.
3. *Intermediaries (firms) are active economic agents* which set the price or rate of resource selling (buying) independently of its stock in such a way that they extract maximum amount of capital. They are similar to a heat engine working fluid in thermodynamics. They can contact with a number of economic agents simultaneously setting different prices and flows for each of them. The intermediary’s prices and its function that describes when to establish/break contact with an economic agent are controls.
  A firm can be a manufacturing firm which buys resources (raw materials, labor, or equipment) and sells its production, which is determined by its production function [1] and the price it sets. We denote the price for the i-th resource set by a firm as $c_{i}$.

### 2.1. Wealth Function and Capital Dissipation

*Existence of a wealth function and its properties*. During an exchange an economic agent sells and buys resources which alters its stocks of resources and capital. Let us introduce the function U by the differential

(2) $$dU=dM+\sum_{i}p_{i}dN_{i}.$$

We shall call it *capitalization* of an economic agent because its variation takes into account changes of capital M as well as changes of *illiquid capital* (stocks) $F=\sum_{i}p_{i}N_{i}$. During equilibrium exchange when prices of selling/buying are infinitesimally close to the estimates $p_{i}$, U does not change, dU=0, as $dM=-\sum_{i}p_{i}dN_{i}$. Such a process is reversible, because the economic agent can buy the same amount of resource as it sold using the capital from the selling and return to the original state without changing anything in its environment.

Suppose equilibrium exchange takes place between a firm and an economic agent when one resource is exchanged for another. Exchange is carried out reversibly and therefore the initial and the final states of the economic agent in the space with coordinates $N_{i}$ coincide. If a firm can extract any capital as a result of this process, then it would be possible to extract an unlimited amount of capital using just one economic agent and not cause any changes in the environment. As this is not possible, it follows that for $p_{0}=\mathrm{const}.$

(3) $$\oint \sum_{i}p_{i}\left(N,M\right)\;dN_{i}=0.$$

From this condition it follows that a function $Z(N,p_{0})$ exists such that its partial derivatives w.r.t. $N_{i}$ are equal to $p_{i}$ and its differential has the form,

(4) $$dZ=\sum_{i}p_{i}\;dN_{i}+\frac{\partial Z}{\partial p_{0}}\;dp_{0}.$$

The condition (2) can be rewritten in the following form,

(5) $$dU=dM+dZ-\frac{\partial Z}{\partial p_{0}}\;dp_{0}=d\left(M+Z\right)-\frac{\partial Z}{\partial p_{0}}\;dp_{0}.$$

After denoting M+Z=Y and $-\frac{\partial Z}{\partial p_{0}}=\gamma$ we get

(6) $$dU=dY+\gamma \;dp_{0}.$$

Thus, this differential is a Pfaffian form with two variables which always has an integrating multiplier.

A *Pfaffian form* is a differential form of degree one, that is, the sum of the products of functions of some variables and the differentials of these variables,

(7) $$dK=\sum_{i=1}^{n}F_{i}\left(x\right)\;dx_{i}.$$

If n=2 and the functions $F_{i}$ are differentiable, then it is always possible to a find multiplier r(x) such that dS=r(x)dK is a complete differential, that is, S depends on x and ∮dS=0. We denote this multiplier for our system $p_{0}\left(N,M\right)$.

Thus, we proved that *there exists a function of state variables (extensive variables), S(N,M), such that its differential has the form*

(8) $$dS=p_{0}\left(N,M\right)dU=p_{0}\left(N,M\right)\left[dM+\sum_{i}p_{i}\left(N,M\right)dN_{i}\right].$$

In a reversible cycle of resource exchange (that is, when the prices of resources coincide with their estimates) the function S does not change,

(9) ∮dS=0.

In cyclic processes the amounts of resources and capital are the same both for initial and final state. The condition (9) is satisfied if the price of some resource and its estimate are equal at each point of the cycle, so resource’s flows are negligible.

The resource estimates can be expressed in terms of the function S as

(10) $$p_{0}=\frac{\partial S}{\partial M},\;p_{i}=\frac{\partial S}{\partial N_{i}}/\frac{\partial S}{\partial M},\;i=1,2,\ldots$$

Here, the capital estimate $p_{0}>0$ for all economic agents, but $p_{i}$ could be negative if the resource requires reprocessing or storage costs.

S(N,M) is called the *wealth function*. The above-described proof of its existence is derived as the consequence of the impossibility to profit indefinitely from trading with one economic agent is an exact copy of the proof of the existence of entropy in thermodynamics. It was obtained by Rozonoer in Appendix of [2]. More general proof is given in [3].

In microeconomics the preferences of an economic agent are often described by its indifference curves (surfaces). Each such curve singles out the set of equally preferred states. If stocks of all resources of an economic agent except one remain constant and this one stock (which could be capital, the basic resource) is increased, then its state is transferred to the higher indifference curve. The existence of S was proven in [4] using the Ville axiom [5], which uses the notion of preferred states of an economic agent: *it is not possible to find a sequence of states $X_{1},X_{2},\ldots ,X_{m}$ in the state space X=(N,M) such that $X_{i}$ is preferred to $X_{i-1}$ for i=2,…,m, and the initial and final states coincide $X_{1}=X_{m}$.*

During resource exchange between economic agents the *voluntary condition*, that the wealth functions $S_{\nu }$ of any participant cannot decrease, must be met (the only exception is exchange associated with charity). The voluntary condition precludes direct exchange of one resource unless its estimates for contacting agents are different. Such exchange becomes possible only if there is an intermediary.

If the wealth function is measured in units of local currency, then $p_{0}>0$ characterizes the value of foreign currency for an agent. Its unit is [unit of local currency/unit of foreign currency]. For currency exchange the estimate $p_{0}$ plays the same role as the estimate p for resource exchange.

The description of economic systems becomes similar to thermodynamic equations if we formally introduce an “economic temperature” as

(11) $$T=\frac{1}{p_{0}}$$

as was done in [2,3].

When the properties of an economic agent do not change but its “scale” changes, its stocks of resources and capital are changed proportionally. It is natural to assume that the wealth function changes in the same way here, that is, that it is an extensive function just like N and M are. In this case, S is a uniform function of first degree and its derivatives on N and M are uniform functions of zero degree. From Euler’s Theorem it follows that it can be written as

(12) $$S\left(N,M\right)=p_{0}\left(M,N\right)\left(\sum_{i}p_{i}\left(M,N\right)N_{i}+M\right).$$

The dependence p(N,M) can be found from experimental data.

If the existence of the wealth function S is postulated, then the estimates may be determined by solving the extremal problem

(13) $$S\left(N,M\right)\to \max/\left(\sum_{i}p_{i}N_{i}+M\right)=V,$$

where V is fixed. In this case, the solution of the problem (13) and the values of p and $p_{0}$ are linked via

(14) $$p_{i}\left(N,M\right)=\frac{\partial S}{\partial N_{i}}/\frac{\partial S}{\partial M}.$$

It is assumed that function S is continuously differentiable and strictly concave. Therefore, the solution of the problem (13) exists and is unique and each $p_{i}$ decreases when $N_{i}$ increases. Thus, an economic agent is similar to a finite capacity subsystem in thermodynamics. For an economic reservoir resource and capital estimates are constant and S is linear.

Despite the similarity of the welfare function to thermodynamic entropy, there are differences between them. In the general case the wealth function is not additive, and the sum of wealth functions for the subsystems is not equal to the wealth function of the entire system. Furthermore, the units of the wealth functions for different subsystems could be different. Unlike the wealth function, the capitalization, the capital, and invested capital have the same unit and are additive.

### 2.2. Differential Links between Estimates—Economic Analogue of the Gibbs–Duhem Relation

Let us write the differential of S

(15) $$dS=p_{0}\left(dM+\sum_{i=1}^{n}p_{i}dN_{i}\right)=p_{0}dU.$$

From (15) we get

(16) $$dM=\frac{dS}{p_{0}}-\sum_{i=1}^{n}p_{i}dN_{i}.$$

From (12) it follows that

(17) $$M=\frac{S}{p_{0}}-\sum_{i=1}^{n}p_{i}N_{i}$$

(18) $$dM=\frac{dS}{p_{0}}+S\;d\left(\frac{1}{p_{0}}\right)-\sum_{i=1}^{n}\left(p_{i}dN_{i}+N_{i}dp_{i}\right).$$

The comparison of (18) and (16) yields the relation between capital estimate and resource estimate,

(19) $$S\;d\left(\frac{1}{p_{0}}\right)-\sum_{i=1}^{n}N_{i}dp_{i}=0.$$

Similarly, comparison of the differential of S found using (12) with the expression (15) yields

(20) $$Mdp_{0}+\sum_{i=1}^{n}N_{i}d\left(p_{0}p_{i}\right)=0.$$

The conditions (19) and (20) follow from the existence of the homogeneous function S. They are economic analogues of the Gibbs–Duhem equation in thermodynamics. One of their consequences when the state of the system is changed in such a way that the resource estimates are constant is that the capital estimate is also constant.

As the matrix of second derivatives for a twice differentiable function is symmetric, the sensitivity of the resource and capital estimates with respect to stock variations are linked by the following equations,

(21) $$\frac{\partial (p_{0}p_{i})}{\partial N_{j}}=\frac{\partial (p_{0}p_{j})}{\partial N_{i}}=\frac{\partial^{2}S}{\partial N_{i}\partial N_{j}}$$

(22) $$\frac{\partial p_{0}}{\partial N_{j}}=\frac{\partial (p_{0}p_{j})}{\partial M}=\frac{\partial^{2}S}{\partial M\partial N_{j}}.$$

It is easy to show using (21) and (22) that

(23) $$\frac{\partial p_{i}}{\partial N_{j}}+p_{i}\frac{\partial p_{j}}{\partial M}=\frac{\partial p_{j}}{\partial N_{i}}+p_{j}\frac{\partial p_{i}}{\partial M},\;i,j=1,\ldots ,n.$$

The Equations (21) and (22) are economic analogs of the Maxwell relations.

### 2.3. Capital Dissipation

Let us again consider the cyclic process of interaction between one economic agent and one intermediary. We now require that the average rate of exchange is fixed. Then, the intermediary has to increase the price above the estimate $p_{i}$ when it is buying and decrease it below estimates when selling. The economic agent’s capitalization here increases because

(24) $$\Delta U=\oint \sum \left(p_{i}\left(N,M\right)-c_{i}\right)\;dN_{i}>0,$$

and the intermediary suffers losses of ΔU in comparison with the reversible process.

The change of capitalization is positive, as $p_{0}\left(N,M\right)>0$.

The rate of the intermediary’s losses due to irreversibility is non-negative,

(25) $$\sigma \left(t\right)=\sum_{i}n_{i}\left(p_{i},c_{i}\right)\left(p_{i}-c_{i}\right)\geq 0.$$

We shall call it the *capital dissipation* due to resource exchange irreversibility. It can be interpreted as trading costs.

The condition (24) of capitalization is non-decreasing (and therefore wealth is non-decreasing) during an economic exchange is analogy to the Clausius integral. The law that during a contact between two economic agents where a resource is transferred from the agent with lower estimate to the agent with higher estimate and that the net invested capital is not decreasing $\left(\Delta \left(F_{1}+F_{2}\right)\geq 0\right)$ is the analogue of of the second law of thermodynamics. It allows us to construct an irreversible microeconomic theory similar to finite-time thermodynamics.

### 2.4. The Second Law of Microeconomics

The resource conservation laws in microeconomics are analogs of conservation of mass and energy in thermodynamics.

Let us consider the economic analogue of the other fundamental law of thermodynamics, the Second Law. Clausius’ statement of the Second Law is “*Heat cannot of itself pass from a colder to a hotter body without some other change, connected herewith, occurring at the same time*.” Leontovich’s formulation is “*It is not possible to build a device which would produce positive work only by cooling one body without any other effects*.”

In microeconomics these formulations correspond to the following statements. (i) *The flux of a scalar resource cannot flow from an economic agent with a higher estimate to an economic agent with a lower estimate without other changes taking place*. (ii) *It is not possible to produce profit by carrying out exchange with one economic agent without any other changes*.

Planck’s statement says “*The entropy of an isolated thermodynamic system during an irreversible process can only increase and its exergy can only decrease. The equilibrium state of such a system has maximum entropy subject to imposed constraints*.”

Similarly, *resource exchange processes in isolated microeconomic systems occur in such a direction that the net capitalization of the economic agents increases and attains a maximum subject to constraints imposed on the system, including the voluntary principle. At the same time, the potential ability to extract profit (profitability) decreases and attains a minimum under the same conditions*.

As the net amount of capital in an isolated system is constant (dM=0), the maximum of capitalization corresponds to maximum of invested capital.

Table 1 shows major analogies between thermodynamic and economic systems. The following notations are used in Table 1. $T_{-}$ and T are temperatures of the reservoir and contacting system, respectively; $p_{-}$ is resource estimate on the perfect competition market; c is the resource price set by an intermediary; N is the stock of resources; U is internal energy; and q and n are the flows of heat and resource, respectively.

## 3. Economic Balances and Capital Dissipation

### 3.1. Open Systems

Consider an open economic system that exchanges resources and capital with an environment. The subscript i denotes the i-th resource and j denotes j-th subsystem. We assume that external flows entering the system are positive and leaving are negative. These flows can be divided into two group. First there are flows caused and effected by external factors. The flows from the second group depend on the prices set by external sellers and buyers and on the estimates of resources in the corresponding subsystem. Similarly to thermodynamics, we shall call the former flows convective and denote them by subscript k and the latter diffusive and denote them by subscript d. Note that a subsystem can produce some resources by using others.

The balance for the i-th resource is

(26) $$\dot{N_{i}}=\sum_{j}\left(n_{ij}^{k}\left(t\right)+n_{ij}^{d}\left(p_{j},c_{j}\right)+W_{j}\left(p_{j}\right)\alpha_{ij}\right),\;i=1,2,\ldots$$

The sum here is over all subsystems, $W_{j}\left(p_{j}\right)$ is the production rate in the j-th subsystem, and the coefficients $\alpha_{ij}>0$ if the i-th resource is produced in the j-th subsystem and $\alpha_{ij}<0$ if it is consumed there. The α’s determine the rate at which the i-th resource is produced (consumed); $c_{j}$ is the price vector for the exchange between the j-th subsystem and its environment.

The balance on capital is

(27) $$\dot{M}=\sum_{j}\left(m_{j}^{k}\left(t\right)-\sum_{i}c_{ij}n_{ij}^{d}\left(p_{j},c_{j}\right)\right).$$

The balance on the invested capital is

(28) $$\dot{U}=\dot{M}+\dot{F}=\dot{M}+\sum_{i,j}p_{ij}\left(N_{j},M_{j}\right)\left(n_{ij}^{k}+n_{ij}^{d}\left(p_{j},c_{j}\right)\right)+\sigma ,$$

where the capital dissipation σ is

(29) $$\sigma =\frac{1}{2}\sum_{j}\sum_{\nu }n_{j\nu }\left(p_{j},p_{\nu }\right)\left(p_{j}-p_{\nu }\right)+\sum_{j}W_{j}\left(p_{j}\right)A_{j}.$$

Here $p_{j}$ and $p_{\nu }$ are vectors of resource estimates for the j-th and ν-th subsystems with the components $p_{ij}$ and $p_{i\nu }$. Correspondingly, $A_{j}=\sum_{i}\alpha_{ij}p_{i}$ and $n_{j\nu }=-n_{\nu j}$ is the vector-function of flow of resources.

The dissipation of capital, similar to the production of entropy in thermodynamics, is calculated as the product of the flow and the driving force, and it is always non-negative. It makes sense of capital losses associated with the creation of a flow of a given intensity. If $\Delta p_{j\nu }=p_{j}-p_{\nu }$ is small and the kinetic function $n_{j\nu }$ is differentiable, then σ is a positive-definite quadratic form.

Just as in the theory of FTT, in economics problems arise about the choice of exchange process parameters when we desire that a given average intensity of exchange flows with a minimum average capital dissipation.

The balance on capitalization is (28) with the capital dissipation σ defined by (29). Here, the first term is due to resource-exchange variation in the amount of illiquid capital and the second is due to production. For profitable production, the dependencies $W_{i}\left(p_{i}\right)$ are such that $W_{i}\left(p_{i}\right)\sum_{j}p_{ij}\alpha_{ij}=W_{i}\left(p_{i}\right)A_{i}\left(p_{i},\alpha_{i}\right)$ are non-negative. $p_{j}$ and $p_{\nu }$ are vectors of resource estimates for contacting subsystems with components $p_{ji}$ and $p_{\nu i}$, and $n_{j\nu }=-n_{\nu j}$.

In a stationary regime the rhs of Equations (26)–(28) are equal to zero. In a cyclic regime when

(30) N(0)=N(τ),M(0)=M(τ),U(0)=U(τ),

the integrals of the rhs of these equations are equal zero.

### 3.2. Isolated Systems

Consider an isolated economic system with no external flows entering or leaving it. Then, the balance Equations (26)–(28) take the form

(31) $${\dot{N}}_{i}=\sum_{j}W_{j}\left(p_{j}\right)\alpha_{ij},\;N_{i}\left(0\right)=\sum_{j}N_{ij}\left(0\right)$$

(32) $$\dot{M}=\sum_{i}{\dot{M}}_{i}=0$$

(33) $$\dot{F}=\sum_{j}{\dot{F}}_{j}=\sigma \geq 0.$$

In equilibrium the invested capital is maximal and the flows $n_{ij}$ and $W_{j}\left(p_{j}\right)$ are equal to zero. The distribution of capital M between the subsystems in equilibrium depends on the kinetics of resource exchange.

For each j-th subsystem of an isolated system

(34) $${\dot{N}}_{ji}=\sum_{\nu }n_{\nu ji}\left(p_{j},p_{\nu }\right)+W_{j}\left(p_{j}\right)\alpha_{ij}$$

(35) $${\dot{M}}_{j}=\sum_{\nu ,i}{\tilde{n}}_{\nu ji}\left(p_{j},c_{j\nu }\right)c_{j\nu i},\;i,j=1,\ldots ,n.$$

Here, $c_{j\nu }$ is the vector of intermediate prices with components $c_{j\nu i}$, which are to be found from the condition of flow continuity,

(36) $${\tilde{n}}_{\nu ji}\left(p_{j},c_{j\nu }\right)=-{\tilde{n}}_{j\nu i}\left(c_{j\nu },p_{j}\right)=n_{\nu ji}\left(p_{j},p_{\nu }\right).$$

Thus, the price vector, and therefore the rhs of equation (35), depends on the forms of kinetic functions of supply and demand, ${\tilde{n}}_{\nu j}$. After expressing $c_{j\nu }$ from (36) and its substitution into ${\tilde{n}}_{j\nu }$ and ${\tilde{n}}_{\nu j}$ both these functions turn out to be equal to the kinetic function $n_{j\nu }\left(p_{j},p_{\nu }\right)$ that was used in (29) and (34).

The equilibrium distribution of capital $\bar{M}$ is determined by the resource exchange kinetics, and $\bar{M}$ depends on equilibrium stocks $\bar{N}$ as they obey the condition

(37) $$p_{j}\left(\bar{M},\bar{N}\right)=p_{\nu }\left(\bar{M},\bar{N}\right)=\lambda ,\;\forall j,\nu .$$

In some cases the object of interest is the set Q of the values of $\bar{M}$ that can be attained from the given initial state for different demand–supply functions $\tilde{n}\left(p,c\right)$.

For each j-th subsystem the minimal capital increase $\Delta M_{j}={\bar{M}}_{j}-M_{j0}$ is achieved when the exchange with all the other subsystems is carried out using $c_{j}$ arbitrarily close to $p_{j}$, that is, reversibly. Therefore, ${\bar{M}}_{j}^{min}$ can be found from the condition $S_{j}\left({\bar{M}}_{j},{\bar{N}}_{j}\right)=S_{j}\left(M_{j0},N_{j0}\right)$.

A maximal ${\bar{M}}_{j}^{max}$ corresponds to such an exchange for which it is possible to construct a range ${\bar{M}}_{j}^{min}\leq M_{j}\leq {\bar{M}}_{j}^{max}$ in the space of $M_{j}$. The intersection of this range with the plane

(38) $$\sum_{j}{\bar{M}}_{j}=\sum_{j}M_{j0}$$

singles out the set Q of all feasible equilibrium distributions of capital.

*As σ(p)>0, the illiquid capital in the flows entering a non-homogeneous open system is always higher than in the flows leaving the system.*

This condition $\sigma (p_{1},p_{2})\geq 0$ jointly with the balances (26)–(28) determine the boundary of the realizability area of irreversible process for an economic systems. The conditions imposed on the rates of various flows allow us to find the minimal capital dissipation, $\sigma_{\min}>0$, achievable for these conditions. This reduces realizability area as the inequality σ≥0 is less restrictive than $\sigma \geq \sigma_{\min}$. This is analogous to the result that the realizability area for a heat engine in FTT is more restricted than if comparison is only made with an equilibrium system.

As the net amount of capital in an isolated system is constant (dM=0), the maximum of capitalization corresponds to the maximum of invested capital.

As an example, we will calculate the capital dissipation σ for exchange between two economic agents with linear kinetics

(39) $$n_{1}\left(p_{1},c\right)=a_{1}\left(p_{1}-c\right),$$

(40) $$n_{2}\left(p_{2},c\right)=a_{2}\left(p_{2}-c\right).$$

From the condition $-n_{1}=n_{2}=n$ we get for $c(p_{1},p_{2})$

(41) $$a_{1}\left(p_{1}-c\right)+a_{2}\left(p_{2}-c\right)=0$$

or

(42) $$c=\frac{a_{1}p_{1}+a_{2}p_{2}}{a_{1}+a_{2}}$$

(43) $$n\left(p_{1},p_{2}\right)=-n_{1}\left(p_{1},c\left(p_{1},p_{2}\right)\right)=\bar{a}\left(p_{2}-p_{1}\right),$$

where

(44) $$\bar{a}=\frac{a_{1}a_{2}}{a_{1}+a_{2}}.$$

The dissipation is then

(45) $$\sigma \left(p_{1},p_{2}\right)=\left(p_{2}-p_{1}\right)\bar{a}\left(p_{2}-p_{1}\right)=\bar{a}{\left(p_{2}-p_{1}\right)}^{2}=\frac{n^{2}\left(p_{1},p_{2}\right)}{\bar{a}}.$$

### 3.3. Maximum Profit Flow

A classical thermodynamic problem is finding the maximum power of a heat engine that receives heat from a source with temperature $T_{+}$ and gives part of it to a source with temperature $T_{-}$, taking into account the irreversibility of heat transfer (see in [6]). The economic analog of this is the problem of finding the maximum profit flow of a company buying an item on the market with an estimate $p_{1}$ and selling it on the market with rating $p_{2}>p_{1}$.

The company must choose the optimal purchase and sales prices $c_{1}$ and $c_{2}$. Let the resource flow be

(46) $$n=k_{1}\left(c_{1}-p_{1}\right)=k_{2}\left(p_{2}-c_{2}\right).$$

Then, after the optimal selection of purchase and sales prices, we get the maximum profit flow

(47) $$m^{*}=\frac{{\left(p_{2}-p_{1}\right)}^{2}}{2A},\;A=\left(1/k_{1}+1/k_{2}\right),$$

the optimal purchase sale flow

(48) $$n^{*}=\frac{p_{2}-p_{1}}{2A},$$

and the corresponding optimal prices

(49) $$c_{1}^{*}=p_{1}+\frac{n^{*}}{K_{1}},\;c_{2}^{*}=p_{2}-\frac{n^{*}}{K_{2}}.$$

## 4. Resource Exchange in Isolated Systems

In isolated economic systems the combined capital does not change $\left(\sum_{j}dM_{j}=0\right)$. Correspondingly, in isolated thermodynamic systems the total energy does not change. Meanwhile, the wealth function and capitalization of each subsystem increase during any resource exchange. This occurs due to increase of combined illiquid capital F, whose differential is

(50) $$dF=\sum_{j}\sum_{i}p_{ji}\left(N_{j},M_{j}\right)dN_{ji}.$$

We will demonstrate later in this paper that when a resource is exchanged for capital (sold) in an isolated system, the combined capitalization of the system,

(51) dU≥0⇒dF≥0

increases. Equality here corresponds to reversible exchange. When one resource is exchanged into another without any exchange of capital (barter) and thus the capital distribution between subsystems is fixed, we have

(52) dF>0.

Therefore, barter is always irreversible (like heat exchange and diffusion processes in thermodynamics).

### Resource/Capital Exchange in Economic Systems with Different Configurations

**Selling.** Suppose the system consists of two economic agents. At t=0 the first economic agent has capital $M_{0}$ and the second holds resource $N_{0}$. At t=0 the estimates obey $p_{1}>p_{2}$, otherwise the trade would be blocked by the voluntary principle.

In equilibrium the balances

(53) $$\bar{M_{1}}+\bar{M_{2}}=M_{0}$$

(54) $$\bar{N_{1}}+\bar{N_{2}}=N_{0}$$

and the equality of estimates

(55) $$p_{1}\left(\bar{N_{1}},\bar{M_{1}}\right)=p_{2}\left(\bar{N_{2}},\bar{M_{2}}\right)=\bar{p}$$

hold. The increase in capitalization of each of economic agents depends on what price is used during exchange. This price must obey the inequality

(56) $$p_{1}\geq c\geq p_{2},$$

otherwise the voluntarity principle would be violated (as the price would be lower than the estimate when resource is sold to economic agent and higher when it is bough from him).

It is clear that

(57) $$\frac{dM_{1}}{dN_{1}}=\frac{dM_{2}}{dN_{2}}=-c,\;dN_{2}=-dN_{1},$$

(58) $$M_{1}\left(0\right)=M_{0},\;M_{2}\left(N_{0}\right)=0,\;N_{2}\left(N_{0}\right)=0.$$

For given $c(N_{1})$ the conditions (57) allow us to express $M_{1},M_{2},N_{2}$ in terms of $N_{1}$. The change in capitalization can then be calculated as

(59) $$\Delta U_{1}=\int_{0}^{\bar{N_{1}}}\left(p_{1}\left(N\right)-c\left(N\right)\right)dN$$

(60) $$\Delta U_{2}=\int_{0}^{\bar{N_{1}}}\left(c\left(N\right)-p_{2}\left(N\right)\right)dN.$$

For the whole system

(61) $$\Delta U=\Delta U_{1}+\Delta U_{2}=\int_{0}^{\bar{N_{1}}}\left(p_{1}\left(N\right)-p_{2}\left(N\right)\right)dN.$$

Because $M_{1},M_{2}$ and $N_{2}$ are expressed in terms of $N_{1}$ and $c(N_{1})$ in (57), $p_{1},p_{2}$ in (59)–(61) depend not only on $N_{1}$.

The conditions (53)–(56) do not fully determine the state of equilibrium. They include three equations for four variables, $\left(\bar{M_{1}},\bar{M_{2}},\bar{N_{1}},\bar{N_{2}}\right)$. When $N_{1}\to \bar{N_{1}}$, the price c tends to $\bar{p}$. If $c=p_{2}$, then $\Delta U_{2}=0$, and the increase of the system’s capitalization is $\Delta U=\Delta U_{1}$. It can be found from Equation (61). If $c=p_{1}$ then $\Delta U=\Delta U_{2}$. The equilibrium states are different in these two cases.

The special case is when c=const and equal to the equilibrium estimate $\bar{p}$. This the case of an *auction*. Here, the price is set in such a way that the amount of bough and sold resource are equal. In this case the condition

(62) $$\bar{M_{2}}=\left(N_{0}-\bar{N_{2}}\right)\bar{p}$$

must be added to the conditions (53)–(55) to determine the final state.

It is not possible to transfer the system from one equilibrium state achieved by choosing some price c, which obeys inequalities (56), into another equilibrium state without reducing its capitalization and wealth function of one of its economic agents. Therefore, the set of equilibrium states is Pareto-optimal (i.e., consists of a set of compromises).

Suppose that the wealth functions have the same dimension. Let us find the state for which the sum $S_{1}\left(\bar{N_{1}},\bar{M_{1}}\right)+S_{2}\left(\bar{N_{2}},\bar{M_{2}}\right)$ attains its maximum subject to constraints (53) and (54). The stationarity conditions of the Lagrange function on the state variables,

(63) $$L=\sum_{i=1}^{2}S_{i}\left(\bar{N_{i}},\bar{M_{i}}\right)+\lambda_{1}\left(\bar{N_{1}}+\bar{N_{2}}\right)+\lambda_{2}\left(\bar{M_{1}}+\bar{M_{2}}\right)$$

leads to the equations

(64) $$\frac{\partial S_{i}}{\partial \bar{N_{i}}}=\lambda_{1},\;\frac{\partial S_{i}}{\partial \bar{M_{i}}}=\lambda_{2},\;i=1,2.$$

As

(65) $$\frac{\partial S_{i}}{\partial \bar{M_{i}}}=p_{i0}\left(\bar{N_{i}},\bar{M_{i}}\right)$$

and

(66) $$\frac{\partial S_{i}}{\partial \bar{N_{i}}}=p_{i0}\left(\bar{N_{i}},\bar{M_{i}}\right)p_{i}\left(\bar{N_{i}},\bar{M_{i}}\right),$$

it follows that in equilibrium, which corresponds to the maximum of the wealth function, both resource estimates $p_{i}$ (see (55)) and the capital estimates $p_{i0}$ are the same for all subsystems. The latter condition makes the set (53)–(55) complete.

If resource estimates do not depend on the capital M, then capitalization U depends on M and N, and dU is a total differential. In this case, it is possible to construct level curves of the function U(N,M) on the plane with coordinates $M_{1},N_{1}$ and origin $O_{1}$. Along these lines, $dU_{1}=0$ and $\frac{dM_{1}}{dN_{1}}=-p_{1}\left(N_{1}\right)$. As the estimate increases when $N_{1}$ increases, the slope of these lines decreases and the curves are convex. The capital $M_{1}\leq M^{0}$. The initial state of the economic agent corresponds to the point $M^{0}$ on the abscissa.

**Figure 1:**
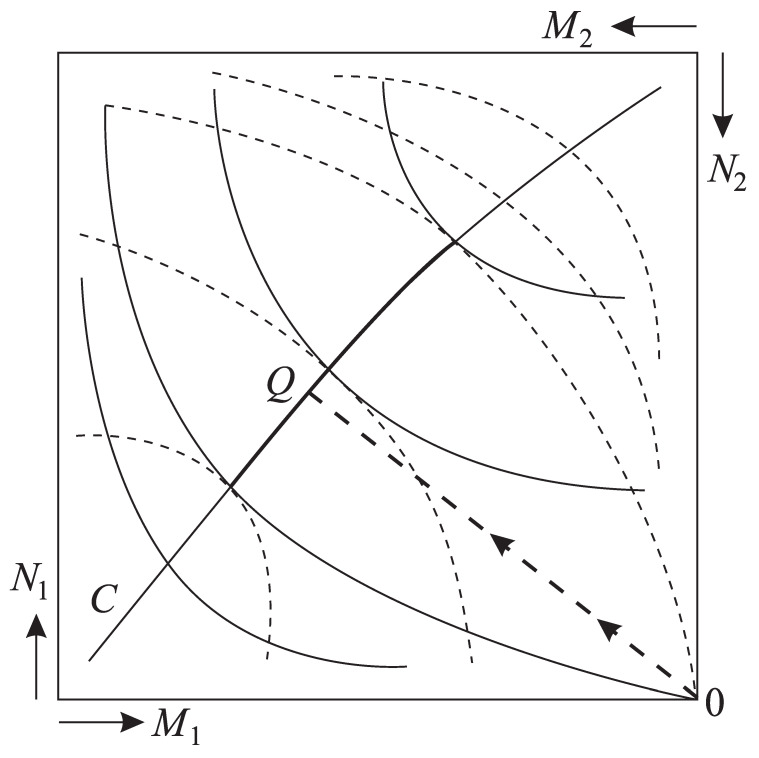
The Edgeworth diagram shows the possibilities of resource exchange in a closed system consisting of two economic agents. The dashed and solid lines show the level lines of the welfare functions of the first and second economic agents, respectively, and the arrows from the origin of the coordinate system in the upper right corner show the directions of growth of resource reserves and capital of each of them. The touch points of the level lines form an equilibrium curve. The section of this curve, highlighted by the bold line, is reachable from the initial state 0, as upon transition to this section the welfare functions of both economic agents increase. The point Q where the vector $\vec{0Q}$ is perpendicular to the bold line corresponds to barter exchange.

Similarly, let us draw the level curves for the capital $M_{2}$ and resource $N_{2}$ of the second economic agent (the origin here is $O_{2}$, the positive direction of the resource $N_{2}$ axis is down, and the capital $M_{2}$ axis is to the left). This figure is called an Edgeworth diagram (see Figure 1). The points where level curves of $U_{1}$ and $U_{2}$ touch obey the conditions of equilibria (55). The set of such points makes the set of equilibrium. The initial state of any system corresponds to the right lower corner of Edgeworth diagram and any of its points obey balances (53) and (54).

However, not all points on the equilibrium curve can be reached without violation of the voluntary condition. Points that can be reached are singled out by the inequality (56). They guarantee that no agent ends up with a lower capitalization. The reachable piece of the equilibrium curve Θ is denoted by the bold line in Figure 1. The point on it which corresponds to auction trading is given by the intersection of this curve with the straight line drawn from the initial state of the system orthogonally to Θ.

Consider exchange through the auction between n economic agents on m kinds of resources occurs. The conditions of equilibrium are

(67) $$p_{i}^{j}\left({\bar{N}}^{i}\right)=\lambda_{j},\begin{array}{c} i=1,\ldots ,n\\ j=1,\ldots ,m. \end{array}$$

We again denote

(68) $$\Delta N_{ij}=\bar{N_{ij}}-N_{ij}\left(0\right),\begin{array}{c} j=1,\ldots ,m\\ i=1,\ldots ,n. \end{array}$$

The balances on resources

(69) $$\sum_{j=1}^{n}\Delta N_{ij}=0,\;j=1,\ldots ,m$$

and capital

(70) $$\Delta M_{j}=-\sum_{i=1}^{m}\lambda_{i}\Delta N_{ij},\;j=1,\ldots ,n$$

need to be added to the conditions of equilibrium (67). The conditions (67)–(70) allow us to find states of all contacting economic agents and the increment of the system wealth function,

(71) $$\Delta S=\sum_{j=1}^{n}\left(S_{j}\left(\bar{M_{j}},\bar{N_{j}}\right)-S_{j}\left(0\right)\right).$$

It is always positive.

For the Cobb–Douglas wealth function,

(72) $$S=M^{\gamma_{0}}\prod_{i=1}^{m}N_{i}^{\gamma_{i}},\;\gamma_{i}\geq 0,\;\sum_{i=0}^{m}\gamma_{i}=1,$$

(73) $$p_{i}=\frac{\partial S/\partial N_{i}}{\partial S/\partial M}=\frac{\gamma_{i}M}{\gamma_{0}N_{i}},\;i=1,\ldots ,m$$

the conditions, derived above, take the form

(74) $$\bar{N_{ij}}=\frac{\gamma_{ij}}{\lambda_{i}}\;\frac{\bar{M_{j}}}{\gamma_{0j}},\;i=1,\ldots ,n,\;j=1,\ldots ,m,$$

(75) $$\bar{M_{j}}=U_{0j}-\sum_{i=1}^{m}\lambda_{i}\Delta N_{ij},$$

(76) $$\lambda_{i}=\frac{\sum_{j=1}^{n}\bar{M_{j}}\frac{\gamma_{ij}}{\gamma_{0j}}}{\sum_{j=1}^{n}N_{ij}\left(0\right)},$$

where $U_{0j}=M_{j}\left(0\right)+\sum_{i=1}^{m}\lambda_{i}N_{ij}\left(0\right)$ is the capitalization of the j-th economic agent with respect to the equilibrium prices.

**Exchange with reservoir**. As a reservoir’s estimates $p^{0}$ are constant, the lines U=const in Figure 1 are straight. Maximal increase of an economic agent’s ΔU corresponds to the exchange using prices $p^{0}$. Then in equilibrium the capital $\bar{M}$ and resource stocks $\bar{N}$ obey the conditions

(77) $$p_{i}\left(\bar{M},\bar{N}\right)=p_{i}^{0},\;i=1,\ldots ,m,$$

(78) $$\bar{M}-M_{0}=\sum_{i=1}^{m}p_{i}^{0}\left(N_{i0}-\bar{N_{i}}\right),$$

(79) $$\bar{M}\geq 0,\;\bar{N_{i}}\geq 0,\;i=1,\ldots ,m.$$

$M_{0}$ and $N_{0}$ are the initial values of M and N. If condition (79) holds, then the conditions (77) and (78) determine the equilibrium state of the system. Otherwise, some of the variables are set to zero which reduces the number of conditions ((77) and (78)) to be used to find the rest of the variables.

Let us calculate the economic agent’s capitalization for m=1.

(80) $$\Delta U=\int_{N_{0}}^{\bar{N}}\frac{dU}{dN}dN=\int_{N_{0}}^{\bar{N}}\left(\frac{\partial U}{\partial M}\;\frac{dM}{dN}+\frac{\partial U}{\partial N}\right)dN.$$

As

(81) $$\frac{dM}{dN}=-p^{0},\;\frac{\partial U}{\partial N}=p\left(M,N\right),\;M=M_{0}-p^{0}\left(N-N_{0}\right)$$

we find

(82) $$\Delta U=\int_{N_{0}}^{\bar{N}}\left[p\left(M_{0}-p^{0}\left(N-N_{0}\right),N\right)-p^{0}\right]dN.$$

If $p\geq p^{0}$ then dN≥0. Otherwise dN≤0 and ΔU is non-negative.

Next, we show that the increase of the economic agent’s wealth function is maximal when market prices are used during the exchange. Indeed,

(83) $$S\left(\bar{N},\bar{M}\right)=S\left(\bar{N_{1}},\ldots ,\bar{N_{m}},M\left(0\right)-\sum_{i=1}^{m}p_{i}^{0}\left(\bar{N_{i}}-N_{i}\left(0\right)\right)\right)\to \max_{\bar{N}}.$$

The conditions of maximum $S(\bar{N})$,

(84) $$\frac{\partial S}{\partial \bar{N_{i}}}=\frac{\partial S}{\partial \bar{M}}\;\frac{\partial \bar{M}}{\partial \bar{N_{i}}}+\frac{\partial S}{\partial \bar{N_{i}}}=p_{0}\left(p_{i}-p_{i}^{0}\right)=0,\;i=1,\ldots ,m$$

coincide with the conditions of equilibrium (77). Thus, the wealth function attains maximum at equilibrium.

Let us specify these equations for the particular case when the economic agent’s wealth function has the Cobb–Douglas form (72). Then, the conditions of equilibrium (77) become the set of linear equations

(85) $$\bar{N_{i}}c_{i}\left(1+\frac{\gamma_{0}}{\gamma_{i}}\right)+\sum_{\nu =1,\nu \neq i}^{m}c_{\nu }\bar{N_{\nu }}=U_{0}=M\left(0\right)+\sum_{\nu =1}^{m}c_{\nu }N_{\nu }\left(0\right),\;i=1,\ldots ,m.$$

Here, $U_{0}$ is capitalization of the economic agent in its initial state using market prices. The solution of Equation (85) becomes

(86) $$\bar{M}=U_{0}\gamma_{0},\;\bar{N_{i}}=U_{0}\frac{\gamma_{i}}{c_{i}},\;i=1,\ldots ,m.$$

The value of the wealth function in equilibrium with the market here is

(87) $$\bar{S}=S\left(\bar{N}\right)=U_{0}\gamma_{0}^{\gamma_{0}}\prod_{i=1}^{m}{\left(\frac{\gamma_{i}}{c_{i}}\right)}^{\gamma_{i}}.$$

**Example** **1.** *Suppose*

(88) $$S=\left(M,N_{1},N_{2}\right)=M^{1/3}\;N_{1}^{1/2}\;N_{2}^{1/6}.$$

*The initial stocks of resources and prices are*

(89) $$M\left(0\right)=1,\;N_{1}\left(0\right)=2,\;N_{2}\left(0\right)=3,\;c_{1}=10,\;c_{2}=20.$$

*The conditions of equilibrium and balance on capital take the form*

(90) $$\frac{\bar{M}}{\bar{N_{1}}}=\frac{20}{3},\;\frac{\bar{M}}{\bar{N_{2}}}=40,$$

(91) $$\bar{M}=1-10\left(\bar{N_{1}}-2\right)-20\left(\bar{N_{2}}-3\right).$$

*From conditions (86) and (87) we obtain*

(92) $$U_{0}=81,\;\bar{N_{1}}=81/20=4.05,\;\bar{N_{2}}=81/120=0.675,\;\bar{M}=27.$$

(93) $$\bar{S}={\bar{M}}^{1/3}\;{\bar{N_{1}}}^{1/2}\;{\bar{N_{2}}}^{1/6}=5.65,\;S\left(0\right)=1.69.$$

*The increase of the economic agent’s wealth function $\Delta S=S\left(\bar{N}\right)-S\left(0\right)=3.96.$*

*For m>1*

(94) $$\Delta U=\sum_{\nu =1}^{m}\int_{N_{0\nu }}^{\bar{N_{\nu }}}\left[p_{\nu }\left(M\left(N\right),N\right)-p_{\nu }^{0}\right]dN_{\nu },$$

*where*

(95) $$M\left(N\right)=M_{0}+\sum_{\nu =1}^{m}p_{\nu }^{0}\left(N_{0\nu }-N_{\nu }\right).$$

**Barter**. The condition that exchange is done voluntarily means that exchange of one kind of resource is possible only if this resource’s estimates by the contacting subsystems have opposite signs. For example, production waste may have negative estimate for one subsystem and positive for the other which can process this waste into useful products. If all estimates have the same sign, then the exchange can only occur if not less than two kinds of resources are exchanged and when there is a counterflow of either capital or another kind of resource (barter). Here, any state for which the vector of resource estimates p for all subsystems are equal, and these resources cannot be used for exchange that would increase the wealth function of the ν-th subsystem,

(96) $$S_{\nu }=p_{0\nu }\left(M_{\nu }+\sum_{i=1}^{n}p_{i}N_{i\nu }\right)=p_{0\nu }U_{\nu }$$

and further they would not reduce the wealth functions of other contacting subsystems, turn out to be equilibrium. Thus, in economics, unlike in thermodynamics, all Pareto-optimal states turn out to be in equilibrium. One of these states corresponds to an exchange via auction. Prices here are determined by the conditions of non-accumulation of resources during re-selling. At the end of the resource exchange, the capitalization $U_{\nu }$ of each subsystem ν based on equilibrium estimates is equal to the initial capitalization. This determines the distribution of capital.

If the functions $S_{\nu }$ have the same dimensionality (which is not always the case), then it is possible to find that state on the Pareto set which maximizes the combined wealth function. This means that none of the subsystems would benefit more from a transition to new new equilibrium state than the others would loose. As we demonstrated above, this state corresponds to equality of the capital estimates,

(97) $$p_{0\nu }=p_{0},\;\nu =1,\ldots ,m,$$

which, jointly with conditions of equilibrium and conditions of non-accumulation, determine the distributions of all resources.

Consider a system that includes two economic agents that both have two types of resources and no capital. In the initial state the resource stocks and their estimates are given by

(98) $$N_{1}^{0}=\left(N_{11}^{0},N_{12}^{0}\right),\;N_{2}^{0}=\left(N_{21}^{0},N_{22}^{0}\right),\;p_{1\nu }^{0}\left(N_{\nu }^{0}\right),\;p_{2\nu }^{0}\left(N_{2}^{0}\right),\;\nu =1,2.$$

The estimates here either do not depend on capital or the distribution of capital $\bar{M}$ is fixed.

If the initial stocks have such values that the solution of the conditions of equilibrium,

(99) $$p_{11}\left(\bar{N_{1}}\right)=p_{21}\left(\bar{N_{2}}\right),$$

(100) $$p_{12}\left(\bar{N_{1}}\right)=p_{22}\left(\bar{N_{2}}\right),$$

(101) $$\bar{N_{1}}+\bar{N_{21}}=N_{11}^{0}+N_{21}^{0},\;\bar{N_{12}}+\bar{N_{22}}=N_{12}^{0}+N_{22}^{0},$$

are positive, then these conditions completely determine the state of the system.

In the general case of barter exchange in which n economic agents take part, each of which holds m kinds of resources, the conditions of equilibrium take the form

(102) $$\sum_{i=1}^{n}\bar{N_{i\nu }}=\sum_{i=1}^{n}N_{i\nu }^{0}=N_{\nu }^{0},\;\nu =1,\ldots ,m,$$

(103) $$p_{i\nu }\left(\bar{N_{i}},\bar{M_{i}}\right)=\lambda_{\nu },\;i=1,\ldots ,n,\;\nu =1,\ldots ,m.$$

For the non-degenerate case of convex (with respect to $\bar{N_{i}}$) functions $p_{i\nu }\left(\bar{N}\right)$, the conditions (102) and (103) determine the equilibrium distribution of resources for fixed capital $\bar{M_{i}}$.

For isolated economic systems the following statement holds.

*For each distribution of initial capital $\bar{M}$ between subsystems, resources are distributed in such a way that the net sum of invested capital attains its maximum conditional on the constraints imposed on the system:*

(104) $$F\left(\bar{M}\right)=\sum_{i}\sum_{\nu }p_{i\nu }\left(N_{i},\bar{M_{i}}\right)N_{i}\to \max_{N_{i}}$$

subject to conditions (102). This maximum is

(105) $$F^{*}\left(\bar{M}\right)=\sum_{\nu }\lambda_{\nu }\left(\bar{M}\right)N_{\nu }^{0}.$$

In its turn the distribution of capital $\bar{M}$ between subsystems obeys the conditions of capital balance, inequalities that follow from the voluntary nature of exchange. It also depends on the form of the kinetic functions.

## 5. Stationary State of an Open Economic System

**Exchange between markets**. Suppose the system consists of two markets. They exchange a vector of resources N whose estimates on the first and second market correspondingly are $p_{1}$ and $p_{2}$. From the economic balances it follows that capital dissipation here is

(106) $$\sigma =\sum_{i}n_{i}\left(p_{1},p_{2}\right)\left(p_{2i}-p_{1i}\right).$$

For flows proportional to the price difference, $n_{i}=a_{i12}\left(p_{2i}-p_{1i}\right)$, analogous to simple flows in thermodynamics, we get

(107) $$\sigma =\sum_{i}a_{i12}{\left(p_{2i}-p_{1i}\right)}^{2}.$$

**Stationary open system**. A stationary regime in an open economic system where there is no convective flows is possible only if it includes at least two economic reservoirs. We denote flows between the ν-th and j-th subsystems as $n_{\nu j}\left(p_{\nu },p_{j}\right)$. For each ν-th economic agent, the vector balances of resources take the following form,

(108) $$\sum_{j}n_{\nu j}+n_{\nu }^{d}+W_{\nu }\alpha_{\nu }=0,$$

where j is the subscript denoting the j-th subsystem. In accordance with (29) capital dissipation becomes

(109) $$\sigma =\frac{1}{2}\sum_{\nu ,j}n_{\nu j}\left(p_{\nu },p_{j}\right)\left(p_{\nu }-p_{j}\right)+\sum_{\nu }n_{\nu }^{e}\left(p_{\nu },c_{\nu }\right)\left(p_{\nu }-c_{\nu }\right)+\sum_{\nu }W_{\nu }\alpha_{\nu }p_{\nu }.$$

In particular, if the system is near equilibrium and flows are proportional to the estimate differences, and the rates of production $W_{\nu j}\alpha_{\nu }$ are constant, then the dissipation takes the following form, similar to exchange between markets,

(110) $$\sigma =\frac{1}{2}\sum_{\nu ,j,i}a_{\nu ji}{\left(p_{\nu i}-p_{ji}\right)}^{2}+\sum_{i,\nu }p_{\nu i}W_{\nu i}\alpha_{\nu i}+\sum_{\nu }a_{\nu i}{\left(p_{\nu i}-c_{\nu i}\right)}^{2}.$$

Here, ν,j are subsystem subscripts and i is the resource subscript.

## 6. Principle of Minimal Capital Dissipation

The factor that causes resource exchange flows to occur (the ”driving force”) is the difference between the resource estimates in two contacting subsystems or between the price and the estimate (for definiteness we will consider the latter). Near equilibrium this difference is small and flows can be assumed to depend linearly on the difference of price and estimate.

The driving force of resource exchange here is Δ=p−c. We assume that the flow directed to the economic agent is positive, then

(111) $$n_{i}=\sum_{\nu =1}^{n}a_{\nu i}\Delta_{\nu }=\sum_{\nu =1}^{n}a_{\nu i}\left(p_{\nu }-c_{\nu }\right),\;i=1,\ldots ,n.$$

We shall call the matrix A with the elements $a_{i\nu }$ the matrix of kinetic coefficients of the economic agent. It determines exchange kinetics between the economic agent and its environment.

The resource exchange flow causes a counter flow of capital such that

(112) $$\frac{dM}{dt}=-\sum_{i=1}^{n}c_{i}n_{i}.$$

The rate of change of the wealth function is

(113) $$\begin{array}{c} \frac{dS}{dt}=\frac{\partial S}{\partial N_{0}}\frac{dN_{0}}{dt}+\sum_{i=1}^{n}\frac{\partial S}{\partial N_{i}}g_{i}=-p_{0}\sum_{i=1}^{n}c_{i}g_{i}++p_{0}\sum_{i=1}^{n}p_{i}g_{i}=\\ =p_{0}\sum_{i=1}^{n}\left(p_{i}-c_{i}\right)g_{i}=p_{0}\Delta^{T}A\Delta . \end{array}$$

Here, Δ is the vector of driving forces.

As capital estimate $p_{0}>0$ and since resource exchange is voluntary and therefore wealth function cannot decrease during an exchange, it follows that the matrix A is positive definite. Let us show that it is also symmetrical.

Indeed, if we extract Δ from (111), then for any infinitesimal time interval the expression (113) takes the form

(114) $$\frac{dS}{p_{0}}=dN^{T}BdN,$$

where dN is the column vector of increments of stocks of resources and $B=A^{-1}$. The elements $b_{i\nu }$ of this matrix are

(115) $$b_{i\nu }=\frac{\partial^{2}\left(\frac{S}{p_{0}}\right)}{\partial N_{i}\partial N_{\nu }}=b_{\nu i},\;i,\nu =1,\ldots ,n.$$

Thus, B is positive definite and symmetric. Therefore, for small deviations from equilibrium, the inverse of the B matrix of supply and demand, $A=B^{-1}$ is symmetric and positive definite. Further, the following *reciprocity conditions* hold. The influence of the difference between the price and estimate of the ν-th resource on the flow of the i-th resource is the same as the influence of the difference of the price and estimate of the i-th resource on the flow of the ν-th resource.

After taking into account the symmetry of the kinetic coefficient matrix, the conditions of minimum σ (see (110)) with respect to $p_{\nu i}$ (ν=1,…,k) for each economic agent lead to the equations such that

(116) $$\sum_{j}a_{\nu ji}\left(p_{\nu i}-p_{ji}\right)+W_{\nu i}\alpha_{\nu i}+a_{\nu i}\left(p_{\nu i}-c_{\nu i}\right)=0\;\forall_{i},\nu .$$

For linear flows this coincides with resource balance equations for each of the subsystems (108). As a consequence, the following statement is true. *Resources and capital are distributed in equilibrium in an open economic system with near linear laws of resource exchange in such a way that capital dissipation σ is minimal.* This is the analogue of the Prigogine principle in irreversible thermodynamics [7].

## 7. Conclusions

We have shown above that the mathematical descriptions of processes occurring between thermodynamic systems and between economic systems have much in common. The concept of irreversibility of economic transport phenomena is introduced, and the problem of minimum irreversibility for a limited duration, or a given average intensity, of economic processes similar to FTT are considered. These analogies are treated in a large number of studies (see [8,9,10,11] a.o.). As so may concepts are similar, here we rather emphasize the main difference between thermodynamic and economic systems.

In thermodynamics any consequences of energy or mass transport between the subsystems of an isolated system are accompanied by an increase in the total entropy. However, the entropy of one subsystem can decrease while the entropy of another one increases by at least the same amount.

In economics not only the total welfare function grows under similar conditions, but also the welfare function of each of the subsystems according to the condition of voluntarity. Moreover, each flow of resource transport is accompanied by a counter flow of capital transport.

Equations of thermodynamic balances correspond in economics to balance equations for capital, for each kind of resources, and for welfare. The role of dissipation is played by the growth rate of the welfare function (it is non-negative). The constraints must be accompanied by the requirements of non-negativity for the growth rate of the welfare function for each subsystem.

## Figures and Tables

**Table 1:** Analogies between thermodynamic and economic systems.

Thermodynamic System		Economic System	
Name	Notation	Name	Notation
Temperature of a system with finite capacity	T	The reciprocal of capital estimate for EA	$1/p_{0}$
Reservoir (irreversible heat exchange)	$q=\alpha (T-T_{-})$	Monopolistic market	$n=\alpha (p-p_{-})$
Mass	N	Resource stock	N
Finite-capacity system, chemical potential	μ(N)	Economic agent, resource estimate	p(N)
Temperature of the working fluid for heat engine	T(t)	Intermediary, price	c(t)
Free energy	A	Capital	M
Internal energy	E	Capitalization	U
Entropy	S	Wealth function	S
Entropy production	σ	Capital dissipation	σ

## References

[1] Petrov A.A. (1966). Economics, Models, Computational Experiment (Ekonomika, Modeli, Vychislitelnyj Eksperiment).

[2] Rozonoèr L.I. (1973). A Generalized Thermodynamic Approach to Resource Exchange and Allocation. II, III. Autom. Remote Control.

[3] Martinás K., Martinás K., Moreau M. (1995). Irreversible microeconomics. Complex Systems in Natural and Economic Sciences.

[4] Hurwicz L., Richter M.K. (1979). An Integrability Condition with Applications to Utility Theory and Thermodynamics. J. Math. Econ..

[5] Ville J., Newman P.K. (1951). The Existence-Conditions of a Total Utility Function. Rev. Econ. Stud..

[6] Curzon F.L., Ahlborn B. (1975). Efficiency of a Carnot engine at maximum power output. Am. J. Phys..

[7] Nicolis G., Prigogine I. (1977). Self-Organization in Nonequlibrium Systems: From Dissipative Structures to Order through Fluctuations.

[8] Lichnerowicz M. (1970). Un modèle d’èchange èconomique (Èconomie et thermodynamique). Annales de l’I.H.P. Probabilités et Statistiques.

[9] Samuelson P.A. (1972). Maximum Principle in Analytical Economics. Am. Econ. Rev..

[10] Salamon P., Komlos J., Andresen B., Nulton J.D. (1987). A Geometric View of Welfare Gains with Non-instantaneous Adjustment. Math. Soc. Sci..

[11] Samuelson P.A., Enlarged (1983). Foundations of Economic Analysis.

